# Flattening the curve of COVID-19 for medical education in psychiatry and addiction medicine

**DOI:** 10.1177/1039856220946647

**Published:** 2020-08-10

**Authors:** Jeffrey CL Looi, Daniel Bonner, Paul Maguire, Angus Finlay, Philip Keightley, Raj Parige, Michael Tedeschi, Rebecca Reay, Soo-Leng Davis

**Affiliations:** Academic Unit of Psychiatry and Addiction Medicine, Australian National University Medical School, Canberra Hospital, Australia; Academic Unit of Psychiatry and Addiction Medicine, Australian National University Medical School, Canberra Hospital, Australia; Academic Unit of Psychiatry and Addiction Medicine, Australian National University Medical School, Canberra Hospital, Australia; Academic Unit of Psychiatry and Addiction Medicine, Australian National University Medical School, Canberra Hospital, Australia; Academic Unit of Psychiatry and Addiction Medicine, Australian National University Medical School, Canberra Hospital, Australia; Academic Unit of Psychiatry and Addiction Medicine, Australian National University Medical School, Canberra Hospital, Australia; Academic Unit of Psychiatry and Addiction Medicine, Australian National University Medical School, Canberra Hospital, Australia; Academic Unit of Psychiatry and Addiction Medicine, Australian National University Medical School, Canberra Hospital, Australia; Australian National University Medical School, Canberra Hospital Campus, Australia

**Keywords:** medical education, psychiatry, COVID-19, adaptation, lessons

## Abstract

**Objective:**

To describe the context, challenges and responses to COVID-19 public health measures for medical education in psychiatry, with an emphasis on sharing strategies for ongoing COVID-19 challenges.

**Conclusion:**

The rapidity of COVID-19 public health measures instituted in Australia required swift action for medical education to address lockdowns of student clinical placements. The responses included a transition to interim online learning followed by a return to truncated clinical placements renegotiated to conform to public health measures. Adjustment of formative and summative assessment has been necessary. However, further contingencies may emerge depending upon the overall progress of the COVID-19 pandemic.

*Si vis pacem, para bellum* (transl. If you seek peace, prepare for war) – *Publius Vegetius Flavius, De rei militarii*.

The COVID-19 pandemic was addressed by the commencement of social distancing lockdown measures, as part of the Australian response to the pandemic on 17 March 2020. We describe the medical educational response to COVID-19 for psychiatry and addiction medicine teaching in the fourth year of the graduate doctor of medicine and surgery degree at the Australian National University (ANU) Medical School, Australian Capital Territory (ACT).^1^ Within the ACT, the ANU main campus shifted to remote work and study on 26 March 2020. Simultaneously, ANU medical students were temporarily excluded from Territory Health Services campuses. Accordingly, medical students were supported via remote learning. The Medical School faculty for Psychiatry and Addiction Medicine, as essential medical and health professional staff, remained at work for clinical duties, while transitioning to remote work for academic duties. Curriculum, as well as formative and summative examination development, continued remotely. Planning commenced for the return of the medical students to clinical placements on 28 April 2020. Presently, in late July 2020, two of the four terms of placements of medical students have been successfully completed, while the third is finishing. We describe our strategies to address the challenges outlined above, in order to provide a framework of experience to assist medical educators, especially in psychiatry and addiction medicine, in flattening the curve of the COVID-19 pandemic impact for basic medical education.

## Maintaining operational effectiveness

Maintenance of operational readiness was essential. All of our faculty, in common with most medical schools in Australia, are specialist clinicians providing essential psychiatric services. Due to increased mental health service demand during the COVID-19 period, there has been prioritisation of clinical care provided by psychiatrists combined with a rapid shift to delivery of mental health care via telehealth consistent with COVID-19 public health measures. The requirement to provide education for medical students in their final year – the interns of 2021 – was also essential.

Our weekly faculty meetings, supplemented by additional digital contact (email/telephone/video), served as the forum for planning and implementation of the educational responses. Social distancing measures for COVID-19 necessitated a rapid shift to video/teleconferencing. In view of the concurrent emerging videoconferencing cybersecurity concerns,^2^ we rapidly reviewed and sourced secure videoconferencing software that would allow for discussion of confidential clinical matters pertaining to medical education (see Table 1).

**Table 1.** Videoconferencing options

*These are some well-regarded videoconferencing options to consider.* *Please determine if these are suitable for your organisation and circumstances.* • Skype/Skype for Business • Microsoft Teams • Webex • Goto Meeting • Wickr Pro • Signal Consider videoconferencing in the context of advice from: https://www.cyber.gov.au/publications/web-conferencing-security

As faculty shifted to working from home for their academic duties, we developed strategies for ongoing communication and workflows. Change in work patterns was facilitated by our existing departmental structure, comprising a Departmental Head, with Co-Deputy Heads, respectively, for curriculum/student placements and assessment/examinations. Our faculty have been working with these academic leads on medical educational tasks. This existing structure allowed for the contingency of staff being unavailable due to sickness or other reasons, in that the Head/Deputy Heads have prior experience of carrying out each other’s duties in the normal course of annual or other leave. In addition to the weekly videoconference, the Head scheduled video/telephone meetings with all Faculty and Heads/Deputy Heads and liaised with medical school administrative support staff (to date, still working from home due to ANU requirements) for teaching, placements and examination.

Enforced working from home might be regarded as remarkable, given that prior to COVID-19 pandemic measures, most people elected to work on the Medical School site. Our faculty shifted to working from home for academic duties to accommodate the requirements of clinical and university roles. Maintaining social distancing of the essential and limited resource of specialist medical/health professionals was the main consideration. We researched and shared information on working from home, including developing this information for the public to maintain occupational mental health with the Australian Medical Association (AMA) Psychiatrists’ Group^3^ (see referenced Webpage for tips on working from home).

## Curriculum and teaching

Due to the lockdown of student placements, and the resultant need to provide interim education from 26 March to 28 April 2020, we decided to make available to students all the video recordings of the whole year lectures on psychiatry and addiction medicine. These lectures provide a foundational introduction and learning framework for our clinical placements, interactive clinical workshops and learning portfolio tasks.^4^ As a further interim measure, our department sourced and provided guidance on self-learning resources (self-test books) that were made available to students. Our faculty liaised with the mental health and addiction medicine clinical administration, and our clinical health professionals as well as consumer teachers, to maintain lecture and interactive workshop teaching. We continued the delivery of lectures that had not yet been recorded or had been revised, again for remote access for students. The delivery of interactive clinical workshops was continued with recorded case-based learning provided by our tutors developing deidentified/fictionalised case presentations in lieu of student case presentations (as students were excluded from the health service).

## Clinical placements

Psychiatry and addiction medicine skills, essential for students to graduate as interns, cannot be developed by remote learning, as opposed to other more didactic academic areas of medicine. Skills gained by face-to-face patient interaction cannot be 100% replaced by online learning, so it was essential for students to return as soon as possible to clinical placements.

The pre-COVID-19 structure of clinical placements comprised a total of 8 weeks, divided into 4-week placements in two clinical settings, with formative clinical tasks assessed through a mandatory clinical portfolio of activities, as well as a supervisor assessment.^4^ Due to medical student exclusion from health services for 1 month, the ANU Medical School instituted a contingency plan comprising the following: 6-week clinical placements; a 4-week catch-up term; and examinations over a 2-week period. Accordingly, we liaised with the mental health and addiction medicine clinical administrators, as well as individual supervisor psychiatrists, to reconfigure for two 3-week placements based on the existing placement structure. In order to address increased student numbers, we increased student placements with the assistance of psychiatrists in private practice and in sub-specialty areas. Given the truncation of the placement duration and increased clinical service demand during COVID-19, we allowed all the learning portfolio tasks and end of placement supervisor assessments to be optional, with a preference for the supervisor assessments to be completed where possible.

Our faculty worked with our psychiatrist supervisors and health professional colleagues to ensure that COVID-19 health measures were appropriately implemented in placements for medical students, complemented by ANU Medical School mandatory education on personal protective equipment (PPE), hygiene and social distancing. This required a considerable degree of flexibility in the context that mental health and addiction medicine services were providing telehealth and modified services to reduce COVID-19 transmission. For example, there were limitations on the number of people in attendance at face-to-face consultations, based on the distancing requirements, as well as COVID-19 screening measures being applied to all attendees for face-to-face consultations. Separate health service protocols were adhered to for home visits, while virtually all aged care face-to-face visits were ceased. Our faculty continued to monitor, receive feedback and act as required through our weekly departmental videoconference meetings with medical school student administrative support also attending and coordinating with our actions.

## Examinations

Medical student examinations in psychiatry and addiction medicine at the ANU Medical School comprise a written multiple-choice question examination integrated with the other clinical specialties in Year 4 (Women’s Health – Obstetrics and Gynaecology, Acute Care, Senior Medicine and Surgery) and two objective structured clinical examinations (OSCEs). OSCEs comprise assessment of mental state examination from a video recording with viva voce examination and an observed clinical interview of a simulated patient or carer.^4^

During COVID-19 measures, our faculty has continued with the regular workflow and timelines for the preparation of the examinations, including preparing the examination materials and standard setting mediated via the ongoing weekly departmental videoconference meetings, during a designated period for examination preparation.

To date, we have completed preparation of the examination materials and planning of the examinations, pending the progress of the COVID-19 pandemic.

## Known unknowns – the limitations of contingency planning

At the time of writing this paper (late July 2020), Australia has seen the rise of the curve of COVID-19 infections, and stalled easing of social distancing restrictions with a second wave of infection in Victoria, requiring the maintenance of social distance and hygiene. Prior to the development and implementation of an effective vaccine for COVID-19, the new normal will be ongoing vigilance and effective action to prevent or ameliorate outbreaks of infection, including assiduous social distancing measures and hygiene. For the foreseeable future, medical education, like the rest of society, will have to maintain certain disciplines. However, whether COVID-19 flares with a second wave of infection, or a vaccine is developed, or the socioeconomic consequences of COVID-19 public health measures remain known unknowns that are arguably beyond the limit of conceivable planning. In responding to COVID-19, medical educators and students will need to continue to change and adapt where possible, hopefully building upon existing organisational efficiency, personal strengths and resilience, and cohesive collegial and peer relationships. Furthermore, it will be important to evaluate the impact of these changes from the perspective of medical students, their supervisors and faculty members, in order to continuously improve our delivery of medical education during COVID-19.

## Lessons from flattening the curve of COVID-19 impact on psychiatric medical education

The key lessons from our experience with psychiatric medical education include:

1. Adapting existing organisation, roles and workflows, which, of course, is predicated on having developed such structures prior to crises such as COVID-19. It would be challenging, if not impractical, to attempt to set up processes *de novo*.
2. Maintaining working relationships with clinical administrators and clinicians in mental health and addiction medicine services.
3. Pivoting to video- and tele-conferencing while maintaining email communication. This has depended upon carefully sourcing secure videoconferencing software suitable for clinical confidentiality and working from home.
4. Scheduling regular communication.
5. Developing new skills and iterative guidelines for COVID-19-related working from home.
6. Ongoing contingency planning, including building capacity to respond to developments such as reinstatement of social distancing and lockdowns for COVID-19 in the event of further outbreaks.
7. Evaluating the impact of changes to curriculum, teaching, clinical placements and examinations.
8. Remaining cognisant that there are known unknowns – contingencies which we cannot necessarily mitigate due to uncertainties in the future.

## Conclusion

By necessity, organisational, educational, personnel and personal adaptations may now represent the new normal for psychiatric medical education, and at least, provide a foundation of skills and knowledge to build upon to address the future challenges of COVID-19.
